# Myostatin inhibition improves hyperglycemia and hyperphagia in lipodystrophic mice

**DOI:** 10.1186/1753-6561-6-S3-P7

**Published:** 2012-06-01

**Authors:** Tingqing Guo, Nichole Bond, William Jou, Oksana Gavrilova, Alexandra C McPherron

**Affiliations:** 1 Genetics of Development and Disease Branch, NIDDK, NIH, Bethesda, MD, 20892, USA; 2 Mouse Metabolic Core Facility, NIDDK, NIH, Bethesda, MD, 20892, USA

## Background

Skeletal muscle is a key peripheral metabolic tissue: Muscle is responsible for most insulin-stimulated glucose uptake, and muscle insulin resistance is the first defect detectable in the development of type 2 diabetes. Recent studies show that muscle mass is inversely correlated with insulin resistance in humans. The growth factor myostatin (MSTN) is a negative regulator of skeletal muscle size. *Mstn* KO mice or mice expressing a dominant negative activin type receptor type IIB (Acrv2B) specifically in muscle (Muscle-DN mice) have increased muscle mass, reduced adipose mass and improved insulin sensitivity. The improvements in insulin sensitivity could be due to the direct effects of the loss of MSTN in muscle or to the secondary effects of reduced adiposity or both. Our hypothesis is that MSTN inhibition specifically in the muscle can improve hyperglycemia in diabetic mice independent of changes in adiposity. To test this hypothesis, we inhibited MSTN signaling in a diabetic model of generalized lipodystrophy (A-ZIP/F-1 mouse model) to analyze its effects on glucose metabolism separate from effects on adipose mass. The A-ZIP/F-1 fatless mouse is a model of lipodystrophy characterized by the lack of white adipose tissue, diabetes, ectopic lipid accumulation and hyperphagia.

## Materials and methods

MSTN signaling was inhibited in A-ZIP/F-1 mice by crossing them to Muscle-DN mice or by injection of soluble Acrv2B. Blood glucose, insulin, insulin sensitivity, triglyceride levels, and food intake were analyzed. The effects of blood glucose on food intake were examined in AZIP/F-1 mice by administration of Phloridzin.

## Results

The development of hyperglycemia, hyperinsulinemia and lipidemia in A-ZIP/F-1 mice was completely suppressed by blocking MSTN function in muscle. Blocking MSTN in A-ZIP/F-1 mice also prevented hyperphagia without any apparent increase in the adipokine leptin. Blood glucose and food intake were also reduced by pharmacologic MSTN inhibition in diabetic A-ZIP/F-1 mice using a soluble receptor. Decreasing blood glucose in A-ZIP/F-1 mice by administration of Phloridzin did not alter food intake.

## Conclusions

These results show that MSTN inhibition can ameliorate diabetes in mice and that this response does not depend on reducing adipose mass. Furthermore, because energy intake is regulated by the central nervous system, our results suggest that muscle may send an as yet unknown signal to the brain, whether directly or indirectly, to regulate energy intake.

